# Observation of viable alloskin vs xenoskin grafted onto subcutaneous tissue wounds after tangential excision in massive burns

**DOI:** 10.1186/s41038-016-0045-9

**Published:** 2016-05-27

**Authors:** Haibin Zuo, Guodong Song, Wen Shi, Jun Jia, Yonghu Zhang

**Affiliations:** Department of Burns, Jinan Central Hospital Affiliated to Shandong University, 105 Jiefang Road, Jinan, Shandong 250013 PR China

**Keywords:** Burn, Skin grafting, Alloskin, Xenoskin, Subcutaneous tissue

## Abstract

**Background:**

Staged excision and grafting with viable cryopreserved alloskin or fresh pigskin at an early stage is a main strategy for wound management in massive burns. Alloskin is the gold standard of a biological temporary skin substitute, and the main drawback to its wider use is the limited number of donors. In this paper, we compare the use of fresh pigskins to cryopreserved alloskins as temporary skin substitutes on subcutaneous tissue wounds after tangential excision by observing the clinical performances of these grafts in cases of a massive burn.

**Methods:**

We selected six adult massive burn patients undergoing tangential excision and skin grafting on subcutaneous tissue wounds (TESGSTW) at our burn center from January 1, 2003 to December 31, 2013. The general clinical data and survival percentage of skins at postoperative weeks (POWs) 1, 2, and 3 were analyzed. In our clinical practice, we also observed the phenomenon that several viable cryopreserved alloskin or fresh pigskin grafts used as temporary coverage on subcutaneous tissue wounds had long-term survival after repeated desquamation. The macroscopic and histological results of one typical case were also analyzed.

**Results:**

In this study, the first three TESGSTW operations were performed at 2–3, 5–8, and 11–16 days post-injury. The operation areas were 30.3 ± 7.9 % total body surface area (TBSA), 19.0 ± 6.0 % TBSA, and 12.0 ± 1.7 % TBSA, respectively. The survival percentage of the cryopreserved alloskins or fresh pigskins at POWs 1, 2, and 3 were 80.0 ± 10.0 % vs 75.7 ± 5.3 % (*t* = 1.01, *P* = 0.16), 71.2 ± 10.6 % vs 66.4 ± 6.2 % (*t* = 1.09, *P* = 0.30), and 48.7 ± 2.5 % vs 35.0 ± 7.0 % (*t* = 3.83, *P* = 0.03), respectively. The microscopic observation of the survival of alloskins or pigskins in one typical case showed rete ridges and a basilar membrane at the joint of the epidermis and dermis at an early stage; these structures disappeared with extended time post-operation.

**Conclusions:**

From the clinical observations, fresh pigskin and cryopreserved alloskins could be used with equal effectiveness at an early stage (within 2 weeks post-operation) as temporary coverage on massive burns after TESGSTW. After engraftment, several cryopreserved alloskins or fresh pigskins could co-survive in a massive burn patient for an extended amount of time. The co-survival of alloskin and pigskin will provide clues for further research into skin transplantation.

**Electronic supplementary material:**

The online version of this article (doi:10.1186/s41038-016-0045-9) contains supplementary material, which is available to authorized users.

## Background

The use of viable biological skins as temporary skin substitutes to prepare the recipient wound after excision for autografting is a lifesaving procedure in the management of massive burns [1]. The common purpose of viable alloskin and xenoskin is to act as a temporary biological dressing to accelerate the granulation and neovascularization of the excised wound. Given the long history of using alloskin and xenoskin as temporary skin substitutes in burn care, the number of (comparative) clinical trials is surprisingly small [2]. The purpose of this article is to compare the clinical performance, advantages, and disadvantages of using cryopreserved alloskins with fresh pigskins as temporary skin substitutes after tangential excision in a massive burn.

Since the 1970s, fascial excision has been successfully performed in massive burn cases, resulting in decreased hospital stays and mortality and reduced blood loss; these grafts typically take well on the fascia [3]. The disadvantages of fascial excision are lymphedema and contour deformities. After the body-contouring adipose tissue is removed, a very significant deformity results, particularly in the chests of female patients [4].

To better preserve the body contouring and to decrease lymphedema, since 1988, we have researched the use of tangential excision for the management of wounds in massive burns [5]. We perform tangential excisions down to the level of viable adipose subcutaneous tissue; these excisions are then covered by viable cryopreserved alloskins or fresh pigskins for wound bed preparation. Subcutaneous tissue wounds following tangential excision not only contain relatively more bacteria and inflammatory edema fluid but these tissues also have poor blood circulation, which can easily result in the loss of the skin graft [6]. After vascularization of reserved viable adipose tissue, auto-skins (microskin or small stamps of skin) were grafted for permanent healing. When autologous donor skin was unavailable, viable alloskin or pigskin was still used to cover all excised sites.

In our clinical practice, we have observed the phenomenon that several viable, cryopreserved alloskin or fresh pigskin grafts could survive for extended periods of time on the debrided wound and after repeated desquamation. The final clinical outcome and histopathological observation of the long-term survival of cryopreserved alloskins or fresh pigskins post-graft were analyzed in this paper.

From January 1, 2003 to December 31, 2013, six adult massive burn cases receiving tangential excision and skin grafting on subcutaneous tissue wound (TESGSTW) operations were selected, and grafts of viable cryopreserved alloskins or fresh pigskins were used as temporary coverage. The general clinical data we analyzed included the operation times; operation areas; selection of coverage on excision wounds; the survival percentage of the grafted viable skins at postoperative weeks (POWs) 1, 2, and 3; long-term survival of the skin areas; macroscopic observation; distribution of body positions; and survival over at least 8 weeks. In a typical case, microscopic observations were performed using hematoxylin and eosin (H&E), Sirius red, and immunohistochemistry staining of the grafted cryopreserved alloskins and fresh pigskins with long-term survival after TESGSTW.

## Methods

### General clinical data

Six adult massive burn patients (four males, two females) who underwent operation by TESGSTW between January 1, 2003, and December 31, 2013, were selected. After the shock stage, the initial two or three TESGSTW operations were performed on the limbs or trunk and were covered with viable cryopreserved alloskins or fresh pigskins. The areas excised in each operation were between 15 and 30 % total body surface area (TBSA). Viable biological skins were used as temporary skin substitutes to prepare the recipient wound after excision for autografting. Autogenetic small stamps of skin (approximately 0.5 cm × 0.5 cm) or microskins were grafted for permanent healing at 2–4 weeks post-tangential excision.

The viable cryopreserved cadaver skins were obtained from the Weifang Medical College transplant bank (Weifang, Shandong province). All donors were aged 20–50 and of Asian descent. The partial-thickness viable cryopreserved skins were 0.4–0.5 mm thick. After cryopreservation in liquid nitrogen, the whole skins were thawed in sterile physiological saline at 42 °C for 1 h before grafting.

According to our protocol, Yorkshire pigs weighing 50 kg and between 3 and 4 months in age were slaughtered and full-thickness skin was excised in the morning of the operation day. The harvested fresh pigskins were washed with soap and water, and the hairs were shaved with a razor blade. The fresh pigskins were scrubbed with 0.1 % benzalkonium bromide solution for 15 min and povidone-iodine solution for 5 min and were then completely cleaned with sterile physiological saline three times. The subdermal fat tissue and partial dermis were removed using a dermatome, and 0.4–0.6 mm partial-thickness skin was harvested for engraftment.

After limb or trunk tangential excision and hemostasis, all wounds were covered with viable cryopreserved cadaver skins and fresh pigskins. The plane of tangential excision extended down to the level of viable subcutaneous adipose tissue. The analysis of the survival percentage of grafted viable skins at POWs 1, 2, and 3, the survival of the skin areas % TBSA, macroscopic observations, and the distribution of body positions were assessed for at least 8 weeks post-operation.

The survival percentage of grafted viable skins = the areas of the survival grafted skins/areas of the grafted skins × 100 %.

In macroscopic observations, the standard used to indicate the survival of grafted viable skins was that the grafted skin was well-attached to the wound, reddish or mildly purple in color, there were no hematocele or empyema under the skin graft, or the epidermis was separated from the dermis; if the epidermis was well-attached to the underlying dermis, the dermis was not withered.

### Histological observation of one typical case

In a typical case, microscopic observations using H&E, Sirius red, and immunohistochemistry staining of cryopreserved alloskin or fresh pigskin grafts with long-term survival after TESGSTW were also performed.

According to the consent of the ethics committee of Jinan Central Hospital and the patients, biopsies for histological examination were incised from the surviving cryopreserved alloskins at 30, 50, and 96 days post-engraftment. Biopsies for pathologic examination from surviving fresh pigskins were incised at 25 and 71 days post-engraftment. Biopsy of co-surviving alloskin and pigskin grafts was incised on day 53 post-injury, with alloskin 50 days post-engraftment and pigskin 25 days post-engraftment on lower extremities. The junction of co-surviving alloskin and pigskin grafts resulted by one pigskin after another alloskin in two operations. The viable alloskin was grafted on day 3 post-injury and fresh pigskin was grafted on day 28 post-injury on the same wound bed of lower extremity after debridement. These biopsies (approximately 0.5 cm × 1.5 cm) were fixed in 10 % buffered neutral formaldehyde and embedded in paraffin for histological analysis. The tissue sections were stained with H&E and Sirius red (direct red 80) (Sigma-Aldrich, USA) in picric acid. Immunohistochemistry staining was performed for human cytokeratin 5/6 (CK 5/6) (Fuzhou Maixin Biotech, China). All samples were photographed under a light microscope (IX71, Olympus, Japan) to evaluate the dermis degeneration and reestablishment of the epidermis in the surviving skin.

### Statistical analysis

All data were expressed as the mean ± SD. The survival percentage of grafted viable cryopreserved alloskins and fresh pigskins at different POWs were tested using an independent sample *t* test, the Levene test, and the *t* test. All analyses were performed using SPSS software (Version 13.0, SPSS, USA) for Windows. *P* < 0.05 was considered statistically significant.

## Results

### General clinical data

The ages of the selected cases ranged 18–35 years old, with an average age of 24.8 years old. Four patients were male and two were female. The total burn areas and full-thickness skin burn areas were 88.3 ± 5.7 % TBSA and 81.6 ± 7.8 % TBSA, respectively.

### Data from the TESGSTW operation

The TESGSTW operation time (post-injury), areas (% TBSA), selection of wound coverage, the auto-skin grafting time after TESGSTW operation (days), and the selection of auto-skin type grafts after TESGSTW operations are shown in Table 1. There were 15 TESGSTW operations performed in total for the selected six cases at an early stage after burn. The first TESGSTW operations were performed at 2–3 days post-injury in six cases; these operations were performed on the lower extremities in five cases and on the upper extremities in one case. The areas excised in these six operations were 30.3 ± 7.9 % TBSA, which were covered with cryopreserved alloskins in four cases and with fresh pigskins in two cases. The second TESGSTW operations were performed at 5–8 days post-injury in six cases; these operations were performed on the upper extremities and trunk in five cases and on the lower extremities in one case. The areas excised in these six operations were 19.0 ± 6.0 % TBSA, which were covered with cryopreserved alloskins in two cases and fresh pigskins in four cases. The third TESGSTW operations were performed at 11–16 days post-injury in three cases. The operation sites were mainly on the trunk. The excised areas were 12.0 ± 1.7 % TBSA, which were covered with cryopreserved alloskins in two cases and with fresh pigskins in one case. The extremities were usually operated on at an early stage, and the trunk of the body was operated on later.

**Table 1:** The TESGSTW operation time (days post-injury), areas (% TBSA), selection of coverage on wound, the auto-skin-grafted time after TESGSTW operations (days post-injury), and selection of auto-skin type grafted after TESGSTW operations

Times of TESGSTW operations (cases)	Post-injury (days)	Operation sites (cases)	Areas of excised (% TBSA)	Coverage (cases)	The auto-skin grafted time after TESGSTW operations (days)	The auto-skin type grafted after TESGSTW operations (cases)
First (6)	2–3	Lower extremities (5)	30.3 ± 7.9	Cryopreserved alloskins (4), fresh pigskins (2)	27.3 ± 3.8	Microskins (2), small stamps of skin (4)
Second (6)	5–8	Upper lower extremities and (or) trunk (5)	19.0 ± 6.0	Cryopreserved alloskins (2), fresh pigskins (4)	22.0 ± 5.7	Microskins (1), small stamps of skin (5)
Third (3)	11–16	Trunk (3)	12.0 ± 1.7	Cryopreserved alloskins (2), fresh pigskins (1)	15.3 ± 1.5	Small stamps of skin (3)

*TESGSTW* tangential excision and skin grafting on subcutaneous tissue wounds, *TBSA* total body surface area

The auto-skin-grafted time after the first, second, and third TESGSTW operations to replace the grafted cryopreserved alloskins or fresh pigskins were 27.3 ± 3.8 days, 22.0 ± 5.7 days, and 15.3 ± 1.5 days, respectively. After the first TESGSTW operations, the auto-skins were used as microskins in two cases and small stamps of skin were used in four cases to replace the cryopreserved alloskin or fresh pigskin grafts. After the second TESGSTW operations, the auto-skins were used as microskins in one case and small stamps of skin were used in five cases to replace the cryopreserved alloskin or fresh pigskin grafts. After the third TESGSTW operations, small stamps of skin were used in all three cases.

### Percentage of survival-viable skins at different POWs

The percentage of surviving viable skins at POWs 1, 2, and 3 are shown in Table 2. Of these 15 TESGSTW operations in a total of six selected cases, 8 operations were covered with cryopreserved alloskins and 7 operations were covered with fresh pigskins. The areas covered with either cryopreserved alloskins or fresh pigskins were 21.8 ± 10.9 % TBSA and 22.4 ± 8.5 % TBSA, respectively. The survival percentage of the cryopreserved alloskins or fresh pigskins at POWs 1, 2, and 3 were 80.0 ± 10.0 % vs 75.7 ± 5.3 % (*t* = 1.01, *P* = 0.16), 71.2 ± 10.6 % vs 66.4 ± 6.2 % (*t* = 1.09, *P* = 0.30), and 48.7 ± 2.5 % vs 35.0 ± 7.0 % (*t* = 3.83, *P* = 0.03), respectively.

**Table 2:** Percentage of survival skins at POWs 1, 2, and 3

Coverage (times of TESGSTW operations)	Area of coverage (% TBSA)	Percentage of survival skins		
		1 week	2 weeks	3 weeks
Cryopreserved alloskins (8)	21.8 ± 10.9	80.0 ± 10.0	71.2 ± 10.6	48.7 ± 2.5 %
Fresh pigskins (7)	22.4 ± 8.5	75.7 ± 5.3	66.4 ± 6.2	35.0 ± 7.0 %*

*TESGSTW* tangential excision and skin grafting on subcutaneous tissue wounds, *TBSA* total body surface area, *POWs* postoperative weeks

*P < 0.05, as compared with cryopreserved alloskins

### Areas and distribution of cryopreserved alloskins or fresh pigskins with survival time over 8 weeks

The areas of cryopreserved alloskins or fresh pigskins with survival times over 8 weeks were 2.0 ± 0.5 % TBSA and 1.5 ± 0.2 % TBSA, respectively. The positions of the survival skins were mainly on the lower extremities and the ventrum. The grafts appeared to be “island” shaped, with ruddy coloration or depigmentation, and with repeated desquamation.

### Macroscopic and histological observation of one typical case

**Fig. 1:**
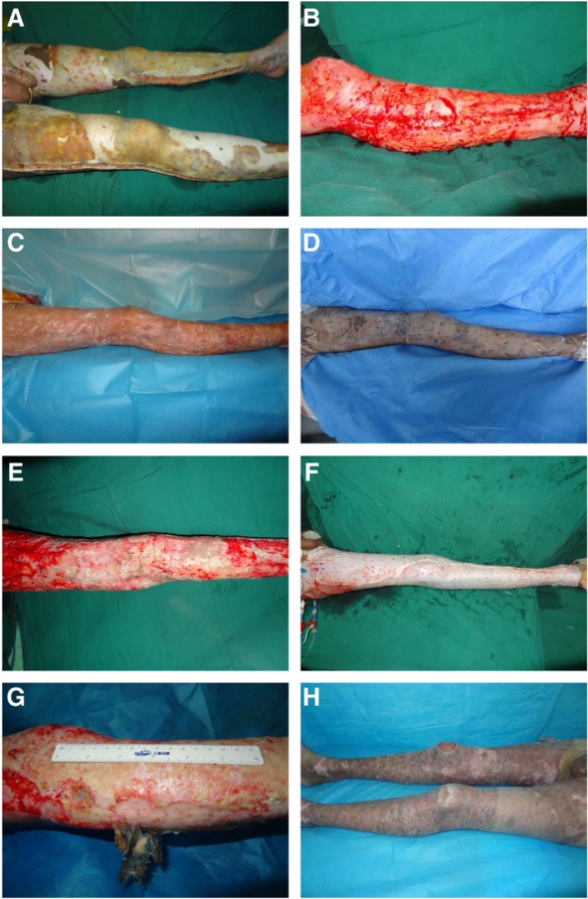
Macroscopic images of the lower extremities in a typical massive burn case. The full-thickness burn wound of the lower extremities is shown in **a**. The wound beds of the lower extremities after the TESGSTW operation and before alloskin grafting are shown in **b**. Macroscopic images of alloskin at POWs 1 and 2 are shown in **c**, **d**, respectively. A macroscopic image of the surviving alloskin on day 30 post-engraftment is shown in **e**, and the grafted fresh pigskin after debridement is shown in **f** and **g** shows the long-term co-survival of alloskin and pigskin grafts on the lower extremities 53 days post-injury, with alloskin 50 days post-engraftment and pigskin 25 days post-engraftment. An image of the healed wounds on the lower extremities at an 8-month follow-up is shown in **h**

Macroscopic images of the lower extremities in a typical massive burn case are shown in Fig. 1. The full-thickness burn wound of the lower extremities and wound bed after the TESGSTW operation is shown in Fig. 1a, b, respectively. Macroscopic images of alloskin at POWs 1 and 2 are shown in Fig. 1c, d. A macroscopic image of the surviving alloskin at 30 days post-graft is shown in Fig. 1e, and the grafted fresh pigskin after debridement is shown in Fig. 1f, and Fig. 1g shows long-term co-survival of alloskin and pigskin grafts on the lower extremities. An image of the healed wounds on the lower extremities at an 8-month follow-up is shown in Fig. 1h.

**Fig. 2:**
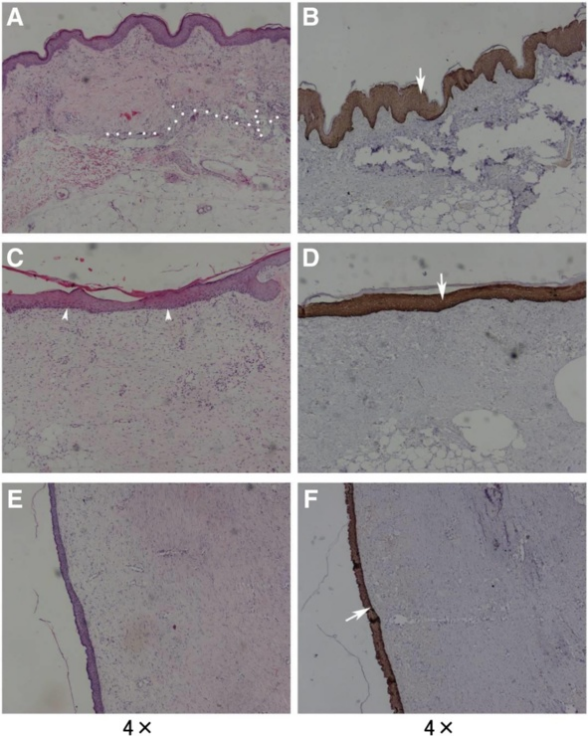
H&E (*left*) and immunochemistry staining (*right*) for human CK 5/6 of surviving cryopreserved alloskins on day 30 (*upper*), 50 (*middle*), and 96 (*bottom*) post-engraftment. The *white dotted line* in **a** shows a band of lymphoid cell infiltration between the recipient’s wound bed and the grafted alloskin. **c** *Arrowheads* show the disappearing structures of rete ridges in the epidermis and the joint of the epidermis and dermis becoming smooth. **b**, **d**, and **f** show immunochemistry staining for human CK 5/6; positive expression (*arrows*) can be observed in the full epidermis

Figure 2 shows the histological observations made in cryopreserved alloskins with long-term survival. A section collected on day 30 was stained with H&E (Fig. 2a). Allogenetic skin with a thickened epidermis that displayed hypergranulosis, prominent lymphocytic inflammatory cell infiltration of the dermis, and abundant adipose tissue under the dermis could be observed. A band of infiltrating lymphoid cells was observed between the recipient’s wound bed and the grafted alloskin, as shown in Fig. 2a. A section collected on day 50 was stained with H&E (Fig. 2c). The rete ridges disappeared into the epidermis, and the joint of the epidermis and dermis (EDJ) was becoming smooth. By 96 days post-engraftment, the degradation of collagen fibers was indicated by the thinning of the collagen bundles of the grafted dermis; these collagen fibers also became more homogenous and were arranged in parallel, as shown in Fig. 2e. The band of lymphocytic inflammatory cells disappeared, and more spindle-shaped fibroblasts infiltrated between the thinner collagen bundles. Immunochemistry staining for human CK 5/6 showed positive expression in the full epidermis, as shown in Fig. 2b, d, f. Figure 2b also shows hypergranulosis in the epidermis of the grafted after 30 days.

**Fig. 3:**
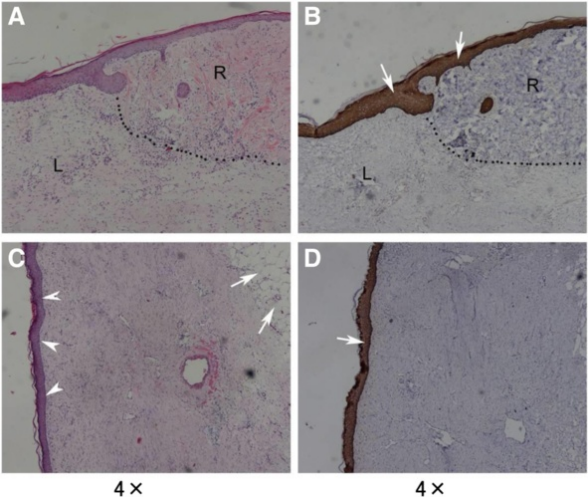
H&E (**a**, **c**) and immunochemistry staining (**b**, **d**) for human CK 5/6 of surviving fresh pigskins on day 25 (**a**, **b**) and 71 (**c**, **d**) post-engraftment. The *black dotted line* shows a sharp dividing line between the pig dermis and the human dermis. **c** shows the pigskin graft on day 71 post-engraftment; the *arrow heads* show the rete ridges disappearing in the epidermis, and the *arrows* show the adipose tissue under the grafted pigskin. **b** and **d** show immunochemistry staining for human CK 5/6 with positive expression (*arrows*) in the full epidermis

A pigskin section stained with H&E (Fig. 3a) also showed hypergranulosis in the epidermis. The black dotted line shown in Fig. 3a, b indicates the junction of the co-surviving alloskin and pigskin grafts on day 53 post-injury, with alloskin 50 days post-engraftment and pigskin 25 days post-engraftment. In Fig. 3a, the pigskin graft is depicted on the right (R) and shows incompletely degraded heavy-stained collagen bundles; lymphocyte migration to the dermis and hypergranulosis in the epidermis can also be seen. To the left (L) of the black line in Fig. 3b is the alloskin graft, which shows incompletely degraded light-stained collagen bundles.

A section collected on day 71 was stained with H&E (Fig. 3c) and showed rete ridges disappearing into the epidermis. The EDJ also became smoother. The collagen bundles of the grafted dermis also became increasingly homogenous, and more fibroblasts migrated to the gaps between the collagen bundles. The adipose tissue under the grafted pigskin is also shown in Fig. 3c. Immunochemistry staining for human CK 5/6 showed positive expression in the full epidermis, as shown in Fig. 3b, d.

**Fig. 4:**
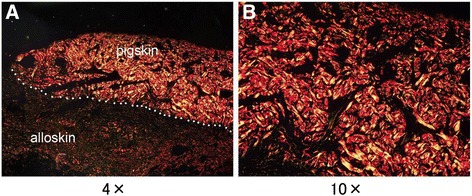
Micrographs showing Sirius red staining of the junction between co-surviving pigskin and alloskin grafts on day 53 post-injury, with alloskin 50 days post-engraftment and pigskin 25 days post-engraftment. The *white dotted line* in **a** shows the sharp dividing line between the pig dermis and the human dermis. **b** shows the thick and birefringent coallgen bundles of survival pigskin dermis on high-power view

Sirius red staining photomicrographs of the junction between pig and human skin on day 53 post-injury, with alloskin 50 days post-engraftment and pigskin 25 days post-engraftment are shown in Fig. 4. Under polarized light, type I collagen appeared as thick fibers with strongly yellow or red birefringence. The grafted pig dermis appeared more strongly birefringent than the adjacent allogenetic dermis under polarized light (Fig. 4a). Figure 4b shows the thick and undegraded pig collagen bundles at a high-power view.

## Discussion

Staged excision and coverage with viable cryopreserved alloskins or fresh pigskins at an early stage is a main strategy for the management of wounds in massive burns [1]. In fascial excision, the skin and subcutaneous tissue are removed, and the wound is debrided to the level of the investing fascia. This approach is used in cases of a massive burn to reduce blood loss or the risk of a severe, life-threatening infection of the subcutaneous tissue [7]. Although the widespread use of fascia excision in a massive burn both decreases hospital stays and mortality, the long-term outcome and quality of life of massive burn survivors are low due to the complications of lymphedema and contour deformities [4]. Thus, in order to enhance the quality of life and improve the mobility of the healed wound, the viable subcutaneous tissue is used to achieve a “bumper” effect. In tangential excision, the viable subcutaneous fat and accompanying lymphatic tissue are preserved, and excision is performed until the level of viable subcutaneous tissue with a yellow glistening appearance is reached. Autografting, such as the use of microskin or small stamps of skin, do not take well on viable adipose tissue before vascularization. However, viable cryopreserved alloskins or fresh pigskins can be used as temporary skin substitutes in order to vascularize the viable adipose tissue.

Since 1988, in order to better preserve the body contours and decrease the incidence of lymphedema, we have researched the use of tangential excision in the management of wounds in massive burn cases [5]. The TESGSTW operation includes tangential excision down to the level of viable adipose tissue, temporarily covering the wound with viable cryopreserved alloskin or fresh pigskin and eventually grafting auto-skin (microskin or small stamps of skin) for permanent healing [6]. This “sandwich technique” (also called the “Meek technique” or the “micrografting technique”) could also be used for wound closure in a massive burn. The use of viable cryopreserved alloskins or fresh pigskin as temporary coverage after excision is common in these techniques before permanent healing of the wound in massive burns, as this strategy uses the characteristics of viable biological skins to “take” on the debrided wound bed. Given the long history of using alloskin and xenoskin as temporary skin substitutes in burn care, the number of comparative clinical trials is surprisingly small [2].

Alloskin is the gold standard of a biological temporary skin substitute that can adhere and become vascularized, establishing vessel-to-vessel connection with the viable wound bed [8, 9]. Cryopreserved alloskins are superior to fresh donor skins in terms of availability, and they provide sufficient time to accurately test the graft for septic contamination [10]. When an alloskin graft adheres to the wound bed and becomes vascularized, as indicated by bleeding upon removal, the wound bed is considered ready for autografting [7, 11]. Cryopreserved alloskin can be vascularized within 3–4 days post-graft and is rejected by the recipient in approximately 2 to 3 weeks, although in rare instances, the alloskin is not rejected and is incorporated into the recipient’s skin as a transplant [12].

Xenoskin, another option for temporary wound coverage, can also become adherent and provides many benefits for the covered wound, such as pain control. Although the underlying wound bed undergoes granulation and neovascularization, the xenoskin itself does not become vascularized. From the early 1960s, a number of burn surgeons started using pigskin as a biological dressing due to limited resources of human alloskin [13]. In many countries, there are no provisions of skin banks for cultural and ethical reasons. Bromberg, in a study of 19 burn patients, reported pigskin to be a suitable replacement for alloskin in terms of the length of time it is used and its adherence [14]. Snyderman studied the survival of homografts and pigskin heterografts in patients with neoplastic disease, observing that pigskin was retained for more than 7 days in 9 out of 10 patients [15]. A study by Brooklyn et al. showed that the pigskin appeared soft and “viable” for nearly 3 weeks (mean survival, 18 days) and then slowly dried and sloughed during the next 2 weeks [14]. In summary, fresh pigskins may become discolored, necrotic, and slough frequently at 10–14 days post-graft, leaving a clean, smooth, granulating wound after being naturally autolyzed or debrided.

Thus, both alloskin and xenoskin are used for temporary wound coverage in excised, non-grafted massive burns. Various observers have attempted to compare the use of alloskin to xenoskin as temporary skin covering. Apparently, there is little difference in the effects of the two procedures to clean a granulating area, prevent water and protein losses, and decrease pain [2].

In this study, the macroscopic observations showed that the reserved viable adipose tissues were glossy, flexible, and had scattered bleeding points at 3–7 days post-injury upon excision. When excised at 7–14 days post-injury, the reserved viable adipose tissues were luteotestaceous, flexible, and had active bleeding. After vascularization of the adipose tissue, the auto-skins were grafted to cover the wounds after excision. In this study, the first, second, and third TESGSTW operations were performed at 2–3, 5–8, and 11–16 days post-injury, respectively, and the areas undergoing the operations were 30.3 ± 7.9 % TBSA, 19.0 ± 6.0 % TBSA, and 12.0 ± 1.7 % TBSA, respectively. The sites that were mainly on the extremities were usually operated on at an early stage (within 2 weeks post-injury), and the trunk of the body was operated on later (within 3–4 weeks post-injury).

To replace the grafted cryopreserved alloskins or fresh pigskins, the auto-skins were grafted after the first, second, and third TESGSTW operations at 27.3 ± 3.8 days, 22.0 ± 5.7 days, and 15.3 ± 1.5 days, respectively. At those times, with debridement of the wounds, the autografts took well on the vascularized viable adipose tissue. The final healed wounds had better body contouring and function, which could be confirmed by microscopic observations of the viable skins that showed long-term survival. The preserved adipose tissues under the grafts are shown in Fig. 2a, b, d.

After alloskin or pigskin grafting, the wounds were repeatedly evaluated either when the dressings were changed or when the patients underwent surgery again. In cases of flaky necrosis or subcutaneous hydrops of the alloskin or pigskin grafts, the grafts were removed in time and replaced and then silver sulfadiazine was applied to the wound. As shown in Fig. 1f, the fresh pigskin was grafted onto the granulated wound for replacement. There was no autologous skin placed underneath.

After vascularization of the adipose tissue, the auto-skins (microskin or small stamps of skin) were grafted to cover the wounds after excision. Macroscopic images of small stamps of auto-skin and scrotum as donor site are shown in Additional file 1: Figure S1. When small stamps of skin were grafted for permanent wound healing, the operative sites were dressed with mesh gauze impregnated with antibiotic ointment, gauzes soaked in antibiotic solution, bulky dressing, and kept in the correct posture. The dressing was first changed 5–7 days post-operation and then once every 2 or 3 days from then on. The grafted autologous microskin was overlain with a sheet of alloskin (microskin grafting) and applied to the subcutaneous tissue wound after the blood circulation had improved [6].

To prolong the survival time of the cryopreserved alloskin or fresh pigskin grafts on the wound bed, hemostasis should be precisely maintained to avoid hematocele formation under the grafted viable skins. The dressings used to fix the grafted skins should be compressed to avoid shifting of the grafts. After the excisions to the viable adipose tissue, viable cryopreserved alloskins were usually chosen for temporary coverage at an early stage (within 2 weeks post-injury) and fresh pigskins were used thereafter.

The transfer of human diseases via allografts, particularly infections with viruses such as HIV, CMV, and hepatitis, is a risk [16–18]. Alloskin availability is also limited by the number of donors. Due to increasingly limited sources of human alloskin, it is necessary to explore suitable, viable temporary coverages in massive burns [19]. Fresh pigskin is cheap and easy to use as a temporary dressing, though it has more antigenicity and a higher rate of infection. In this study, the survival percentages of cryopreserved alloskin and fresh pigskin grafts at POWs 1 and 2 showed no significant differences. At nearly 3 weeks after TESGSTW, the cryopreserved alloskin or fresh pigskin grafts were rejected or removed for immunological rejection and the auto-skin transplantation operations were performed.

The clinical outcomes of grafted viable human or pigskins as temporary substitutes could be summarized in three ways: dissolution, necrosis, and desquamation. Dissolution of grafted alloskin or pigskin resulted in wound reexposure, enhancing opportunities for infection. With living and expending of grafted autologous microskin or small stamps of skin after Meek grafting or micrografting, the blood supply of alloskin or pigskin will be blocked and result in necrosis. Desquamation is the best type of clinical outcome of grafted skins in terms of wound healing. The phenomenon of repeated desquamation in the cryopreserved alloskin or fresh pigskin grafts has been termed “creeping substitution” by Brown and McDowell [20]. This phenomenon has also been defined as “desquamation healing” by Zhang in China [21]. In this study, the areas of cryopreserved alloskins or fresh pigskins with survival times of over 8 weeks were 2.0 ± 0.5 % TBSA and 1.5 ± 0.2 % TBSA, respectively. The positions of the surviving skins were mainly on the lower extremities and the ventrum, and the grafts appeared to be “island” shaped, with ruddy coloring or depigmentation, and with repeated desquamation.

The microscopic observation of the grafted human and pigskins by H&E staining showed that both epidermises had rete ridges. Over time, the EDJ became smoother, the rete ridges were lost, and lymphocytes and fibroblasts migrated into the gaps between collagen bundles in both graft types. Moreover, the papillary and upper reticular dermis was replaced with dense, relatively avascular homogenized collagen. In 2006, the pathology working group of the National Institute of Health Consensus Development presented the progression of histological changes from acute to chronic cutaneous graft-versus-host disease (GVHD) [22]. The minimal criterion for the diagnosis of cutaneous sclerotic chronic GVHD is the homogenization of most of the papillary dermis or reticular dermis. The skin can also display a combination of residual damage, with the loss of rete ridges and dermal appendages as well as some increases in papillary or dermal sclerosis after immunosuppressive treatment [23].

At the junctions between grafted human and pigskins that had co-survived long term, we could observe that the pig dermis had thick and heavy-stained collagen bundles compared to the grafted human dermis at an early stage. The Sirius red staining showed an obvious junction between the alloskins and pigskin. Immunohistochemistry of the grafts showed positive expression of human CK 5/6 in the reestablished epidermises of the alloskins and pigskins. Whether the origin of the reestablished epidermis in the human and pigskin grafts with long-term survival is actually the patient can be determined by DNA identification in future research. The results will provide clues for further research of xenogenetic or allogenic skin transplantation.

For allogenic or xenogenic skin transplantation, immunogenicity is a major obstacle to prolonged use. For decreased immunological rejection of the grafted fresh pigskins, some pretreatments can be performed, such as ultraviolet irradiation [24] or soaking in fluocinolone acetonide. The extracorporeal photochemotherapy (ECPCT) approach has a complementary effect on preventing rejections with the absence of the known side effects of conventional immunosuppression [25]. Pigs engineered to lack the gene for α-1,3-galactosyltransferase (GTKO) became available for experimental studies in 2002 [26]. The results of preclinical transplantation of GTKO pig cells or corneas are much more encouraging than those for organ transplantation, with survival times of greater than 1 year [27]. Whether this is also the case when the fresh skin of GTKO pigs is used instead of alloskin as a skin temporary substitute remains unclear. We can explore the use of engineered pigskins as temporary substitutes to reduce immunological rejection and prolong the survival time.

## Conclusions

From the clinical observations, fresh pigskin was found to be as effective as cryopreserved alloskins for use in temporary coverage after TESGSTW for massive burns. In the absence of available cadaver alloskin, fresh pigskin is a satisfactory substitute for use as a temporary coverage. The co-survival of alloskin and pigskin will provide clues for further research into allogenic and xenogenic skin transplantation.

## Additional file

Additional file 1:
**Figure S1.** Macroscopic images of small stamps of auto-skin and scrotum as donor site. Small stamps of auto-skin (approx. 0.5 cm×0.5 cm) for permanent healing are shown in **a**, and the use of scrotum as a donor site of a typical massive burn case is shown in **b**. (DOC 94 kb)
