# Supplementary material for: Observation of viable alloskin vs xenoskin grafted onto subcutaneous tissue wounds after tangential excision in massive burns
Source: Burns Trauma. 2016 May 27;4:23. doi: 10.1186/s41038-016-0045-9 (PMC4964051; doi:10.1186/s41038-016-0045-9)
Supplement: Additional file 1: — Figure S1. Macroscopic images of small stamps of auto-skin and scrotum as donor site. Small stamps of auto-skin (approx. 0.5 cm×0.5 cm) for permanent healing are shown in a, and the use of scrotum as a donor site of a typical massive burn case is shown in b. (DOC 94 kb) [file 41038_2016_45_MOESM1_ESM.doc]

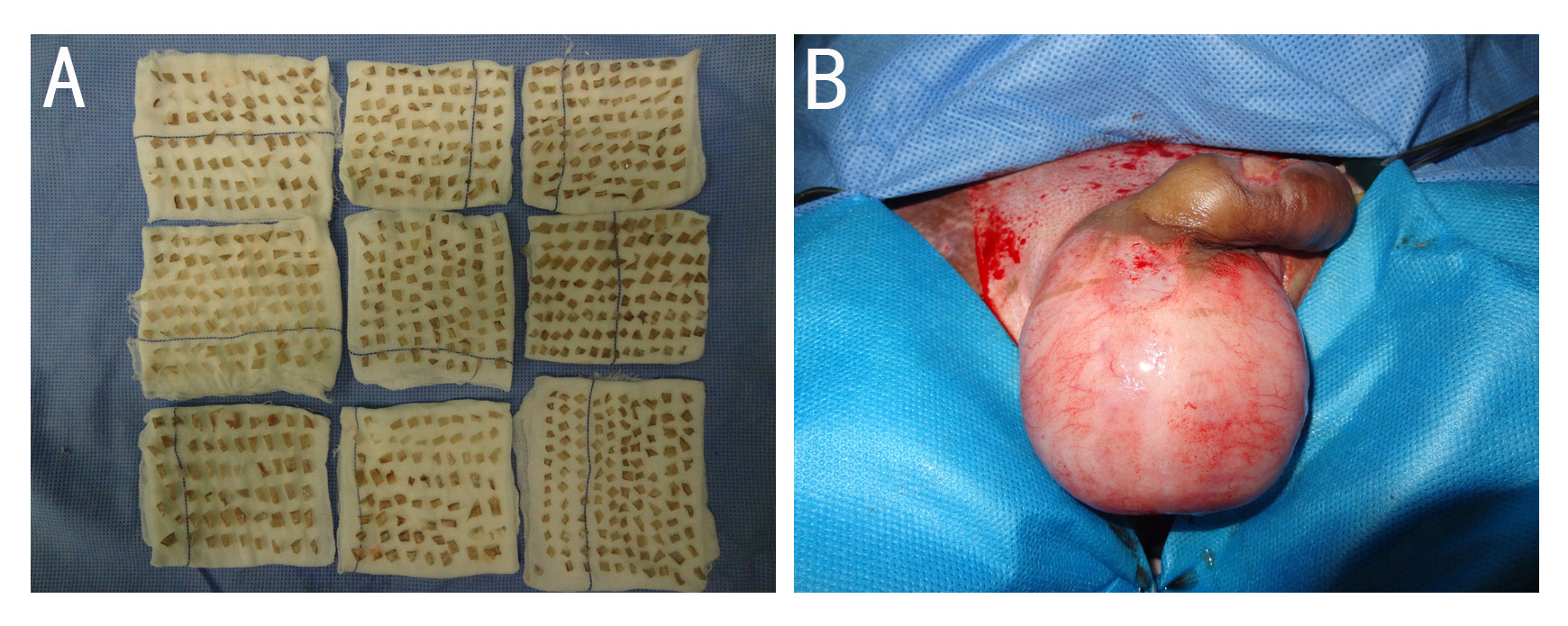


**Figure S1** Macroscopic images of small stamps of auto-skin and scrotum as donor site. Small stamps of auto-skin (approx. 0.5 cm×0.5 cm) for permanent healing are shown in **a**, and the use of scrotum as a donor site of a typical massive burn case is shown in **b**.
